# Short-Term Response of Cytosolic $\text{N}{\text{O}}_{3}^{-}$ to Inorganic Carbon Increase in *Posidonia oceanica* Leaf Cells

**DOI:** 10.3389/fpls.2020.00955

**Published:** 2020-06-25

**Authors:** Lourdes Rubio, Delia García-Pérez, Julia M. Davies, José A. Fernández

**Affiliations:** ^1^ Departamento de Botánica y Fisiología Vegetal, Facultad de Ciencias, Universidad de Málaga, Málaga, Spain; ^2^ Department of Plant Sciences, University of Cambridge, Cambridge, United Kingdom

**Keywords:** cytosolic NO_3_^−^, elevated inorganic carbon, NO_3_^−^ efflux, anion channels, intracellular nitrate-selective microelectrodes, seagrasses

## Abstract

The concentration of CO_2_ in the atmosphere has increased over the past 200 years and is expected to continue rising in the next 50 years at a rate of 3 ppm·year^−1^. This increase has led to a decrease in seawater pH that has changed inorganic carbon chemical speciation, increasing the dissolved $\text{HC}{\text{O}}_{3}^{-}$. *Posidonia oceanica* is a marine angiosperm that uses $\text{HC}{\text{O}}_{3}^{-}$ as an inorganic carbon source for photosynthesis. An important side effect of the direct uptake of $\text{HC}{\text{O}}_{3}^{-}$ is the diminution of cytosolic Cl^−^ (Cl^−^c) in mesophyll leaf cells due to the efflux through anion channels and, probably, to intracellular compartmentalization. Since anion channels are also permeable to $\text{N}{\text{O}}_{3}^{-}$ we hypothesize that high $\text{HC}{\text{O}}_{3}^{-}$, or even CO_2_, would also promote a decrease of cytosolic $\text{N}{\text{O}}_{3}^{-}$ ($\text{N}{\text{O}}_{3}^{-}{}_{\text{c}}$). In this work we have used $\text{N}{\text{O}}_{3}^{-}$- and Cl^−^-selective microelectrodes for the continuous monitoring of the cytosolic concentration of both anions in *P. oceanica* leaf cells. Under light conditions, mesophyll leaf cells showed a $\text{N}{\text{O}}_{3}^{-}{}_{\text{c}}$ of 5.7 ± 0.2 mM, which rose up to 7.2 ± 0.6 mM after 30 min in the dark. The enrichment of natural seawater (NSW) with 3 mM NaHCO_3_ caused both a $\text{N}{\text{O}}_{3}^{-}{}_{\text{c}}$ decrease of 1 ± 0.04 mM and a ${\mathrm{Cl}}_{\text{c}}^{-}$ decrease of 3.5 ± 0.1 mM. The saturation of NSW with 1000 ppm CO_2_ also produced a diminution of the $\text{N}{\text{O}}_{3}^{-}{}_{\text{c}}$, but lower (0.4 ± 0.07 mM). These results indicate that the rise of dissolved inorganic carbon ($\text{HC}{\text{O}}_{3}^{-}$ or CO_2_) in NSW would have an effect on the cytosolic anion homeostasis mechanisms in *P. oceanica* leaf cells. In the presence of 0.1 mM ethoxyzolamide, the plasma membrane-permeable carbonic anhydrase inhibitor, the CO_2_-induced cytosolic $\text{N}{\text{O}}_{3}^{-}$ diminution was much lower (0.1 ± 0.08 mM), pointing to $\text{HC}{\text{O}}_{3}^{-}$ as the inorganic carbon species that causes the cytosolic $\text{N}{\text{O}}_{3}^{-}$ leak. The incubation of *P. oceanica* leaf pieces in 3 mM $\text{HC}{\text{O}}_{3}^{-}$-enriched NSW triggered a short-term external $\text{N}{\text{O}}_{3}^{-}$ net concentration increase consistent with the $\text{N}{\text{O}}_{3}^{-}{}_{\text{c}}$ leak. As a consequence, the cytosolic $\text{N}{\text{O}}_{3}^{-}$ diminution induced in high inorganic carbon could result in both the decrease of metabolic N flux and the concomitant biomass N impoverishment in *P. oceanica* and, probably, in other aquatic plants.

## Introduction

Nitrate ion ($\text{N}{\text{O}}_{3}^{-}$) is the main source of inorganic nitrogen for plants in aerobic conditions. Compared with the concentrations in soils, the concentration of $\text{N}{\text{O}}_{3}^{-}$ in seawater is persistently very low, particularly in the oligotrophic Mediterranean Sea (Lepoint et al., 2002; Bethoux et al., 2005). Seagrasses are the unique angiosperms that evolved from land plants to live submerged in the sea, forming the basis of the most productive and widespread coastal ecosystems on the planet (Larkum et al., 2006). For vascular plants, colonizing the sea implicates losses and gains to effect structural and physiological adaptations to complete the submerged life cycle, achieved by a reverse evolutionary trajectory in the seagrass lineage (Williams, 2016). Thus, key land angiosperm innovations were lost in seagrasses including the entire collection of genes involved in stomata differentiation, genes related to the synthesis and sensing of terpenoids and other volatile substances, genes for ultraviolet protection, and phytochromes for far-red sensing (Olsen et al., 2016). However, to survive in the conditions of low $\text{N}{\text{O}}_{3}^{-}$ availability, seagrasses have evolved high affinity uptake systems to capture $\text{N}{\text{O}}_{3}^{-}$ through their leaves. These systems have been characterized for *Zostera marina* in which the uptake of $\text{N}{\text{O}}_{3}^{-}$ and inorganic phosphate (Pi) are driven by the inwardly directed electrochemical gradient for Na^+^ (García-Sánchez et al., 2000; Rubio et al., 2005). In the Mediterranean, *Posidonia oceanica* is an endemic coastal species of huge ecological importance (Aires et al., 2011). Similar high-affinity and Na^+^-dependent uptake mechanisms also operate in *P. oceanica* for both nutrients and some amino acids (Rubio et al., 2018). In both cases, the low semi-saturation constants observed (2.3 and 8.7 µM $\text{N}{\text{O}}_{3}^{-}$ for *Z. marina* and *P. oceanica*, respectively: García-Sánchez et al., 2000; Rubio et al., 2018) indicate that those systems are very efficient for $\text{N}{\text{O}}_{3}^{-}$ uptake at the very low concentrations of $\text{N}{\text{O}}_{3}^{-}$ in seagrass meadows (Touchette and Burkholder, 2000; Romero et al., 2006). Nevertheless, those systems are energetically expensive because seagrass leaf cells have to keep low homeostatic Na^+^ concentrations in the cytosol to maintain the Na^+^ motive force (Rubio et al., 2011; Rubio et al., 2018). In this scenario, namely low availability of $\text{N}{\text{O}}_{3}^{-}$ and the energetically expensive mechanism for its high-affinity uptake, the maintenance of $\text{N}{\text{O}}_{3}^{-}$ inside the cells appears critical. Therefore, as with other vascular plants, seagrasses must maintain intracellular $\text{N}{\text{O}}_{3}^{-}$ homeostasis to preserve the N metabolic flux.

In a previous work, besides cytosolic H^+^ and Na^+^, we also measured cytosolic Cl^−^ (using intracellular ion-selective microelectrodes) in the mesophyll cells of *P. oceanica* (Rubio et al., 2017). In that work, we demonstrated that this seagrass has a direct plasma membrane symporter to uptake $\text{HC}{\text{O}}_{3}^{-}$ driven by the H^+^ electrochemical potential gradient. A significant increase of photosynthesis in natural seawater supplemented by 3 mM $\text{HC}{\text{O}}_{3}^{-}$ supported the role of $\text{HC}{\text{O}}_{3}^{-}$ uptake in the photosynthetic activity of this species (Rubio et al., 2017). Furthermore, the enrichment of seawater with 3 mM $\text{HC}{\text{O}}_{3}^{-}$ also evoked a delayed, but significant, diminution of the cytosolic Cl^−^ concentration (Rubio et al., 2017). Similar cytosolic Cl^−^ efflux has been observed in guard cells of the model land angiosperm *Arabidopsis thaliana* during stomatal closure in response to elevated CO_2_ (Xue et al., 2011). This Cl^−^ efflux from guard cells is described as taking place through the plasma membrane S-type anion channels, whose activation responded to the cytosolic $\text{HC}{\text{O}}_{3}^{-}$ concentration (Xue et al., 2011). Interestingly, in addition to Cl^−^ these anion channels have a high permeability to $\text{N}{\text{O}}_{3}^{-}$ (Schmidt and Schroeder, 1994), which is also released from the guard cells during stomatal closure (Geiger et al., 2011; Demir et al., 2013; Maierhofer et al., 2014).

The release of Cl^−^ from *P. oceanica* in response to $\text{HC}{\text{O}}_{3}^{-}$ is driven by the outwardly directed electrochemical potential gradient for Cl^−^ (Rubio et al., 2017). Considering Cl^−^-selective microelectrodes are also partially sensitive to $\text{N}{\text{O}}_{3}^{-}$ (Miller and Zhen, 1991) we hypothesize that the increase of inorganic carbon in seawater could also lead to the decrease of cytosolic $\text{N}{\text{O}}_{3}^{-}$ in *P. oceanica* leaf cells. Such diminution would affect assimilation (Bloom et al., 2010) and could partially be responsible for the plant biomass nitrogen impoverishment expected under elevated inorganic carbon in vascular plants with non-saturated photosynthesis (Taub and Wang, 2008).

Therefore, the aim of this work was to measure the cytosolic $\text{N}{\text{O}}_{3}^{-}$ concentration in mesophyll leaf cells of *P. oceanica* to describe the responses to light and dark conditions and to the increase of dissolved inorganic carbon (CO_2_ and $\text{HC}{\text{O}}_{3}^{-}$) in natural seawater. Ethoxyzolamide, the plasma membrane-permeable carbonic anhydrase inhibitor (Sültemeyer et al., 1993), has been used in combination with CO_2_ to test for the effect of limiting any CO_2_-dependent $\text{HC}{\text{O}}_{3}^{-}$ generation. Since the first work in *Chara corallina* (Miller and Zhen, 1991), $\text{N}{\text{O}}_{3}^{-}$-selective microelectrodes have been used in different plant species, such as barley (Zhen et al., 1991; Van Der Leij et al., 1998), *A. thaliana* (Cookson et al., 2005) and rice (Fan et al., 2007), plus the liverwort *Conocephalum conicum* (Trębacz et al., 1994). However, so far no direct measurement of cytosolic $\text{N}{\text{O}}_{3}^{-}$ has been reported for marine vascular plants. Furthermore, external $\text{N}{\text{O}}_{3}^{-}$ has been monitored, and the potential effects of elevated atmospheric CO_2_ on the hypothesized diminution on the biomass nitrogen content are also discussed.

## Materials and Methods

### Plant Material

*Posidonia oceanica* (L.) Delile plants were sampled in Punta de Calaburras (36°30′23.4′′N 4°38′37.6′′W) Málaga, southern Spain, at 2 m depth. Plants with 6 to 12 leaves attached to a piece of the rhizome were collected and transported to the laboratory in a thermos container in less than 30 min. Then, plants were placed in an aquarium filled with continuously aerated natural seawater (NSW). The air used for this purpose was obtained from the compressed air supply of the Faculty of Sciences building. Concentration of CO_2_ in the air was regularly monitored (390 ± 10 ppm) using an IRGA, LICOR LI-820, Li, Nebraska (USA). Temperature was held at 15°C, and illumination was at a light intensity of 150 µmol photons·m^−2^·s^−1^ with a photoperiod 16L/8D. Renewing the seawater every three days, plants were used for experiments within two weeks after sampling.

### Cytosolic $\text{N}{\text{O}}_{3}^{-}$ and Cl^−^ Measurements

Cytosolic nitrate and chloride were measured by electrophysiological techniques using double-barreled intracellular microelectrodes. Glass capillary preparation details have been previously described (Fernández et al., 1999). In short, double-barreled capillaries with different diameter (1.5 and 0.75 mm o.d., respectively, Hilgenberg, Germany) were twisted before pulling using a Narishige PD-5 horizontal puller. Then, pulled double-barreled capillaries were heated for 30 min at 180°C and silanized by adding one drop of dichlorodimethylsilane dissolved in benzene (0.05% v/v) to the interior of the blunt end of the larger barrel, while the smaller barrel (that operates as voltage electrode) was not silanized. After that, the silanized double-barrelled capillaries were heated again for 60 min at 180°C. Once cool, the silanized barrel was backfilled with the appropriate chloride or nitrate sensor solution.

For Cl^−^-microelectrodes, the ionophore I (99408, Fluka), dissolved in a mixture of polyvinylchloride dissolved in tetrahydrofuran (PVC/THF, 4% w/v) was used and backfilled into the silanized barrel. Once THF evaporated, the remainder was filled with 0.5 M KCl (Planes et al., 2015). As described previously (Rubio et al., 2017), Cl^−^-microelectrodes were calibrated against NaCl solutions (1–100 mM) that contained 5 mM NaNO_3_, the putative cytosolic $\text{N}{\text{O}}_{3}^{-}$ concentration (Miller and Smith, 2008), to minimize the interference between Cl^−^ and $\text{N}{\text{O}}_{3}^{-}$ in the cytosol (Felle, 1994). Calibration showed a linear relationship of 37 mV/pCl.

$\text{N}{\text{O}}_{3}^{-}$-microelectrodes were backfilled using a $\text{N}{\text{O}}_{3}^{-}$ sensor (Fluka 72549) based on the quaternary ammonium compound, methyltridodecylammonium nitrate (MTDDA.$\text{N}{\text{O}}_{3}^{-}$) containing the PVC/THF solution. These liquid ion-exchange based microelectrodes are highly selective for $\text{N}{\text{O}}_{3}^{-}$, maintaining a nitrate detection limit of 0.5 mM in the presence of 100 mM Cl^−^, which means any interference will not be important in a physiological situation, as described in Miller and Zhen (1991). Once THF was evaporated from the nitrate-sensor cocktail and before use, the $\text{N}{\text{O}}_{3}^{-}$-selective barrel was backfilled with 0.1 M NaNO_3_ and 0.1 M KCl. Then, $\text{N}{\text{O}}_{3}^{-}$-selective electrodes were calibrated against NO_3_K solutions (0.1–20 mM) containing 10 mM KCl, to saturate the presumed interference using the Cl^−^ concentration reported previously in *P. oceanica* mesophyll leaf cells (Rubio et al., 2017), and KH_2_PO_4_ (from 15 to 50 mM), to give a constant background ionic strength in the calibration solutions (Miller and Zhen, 1991). $\text{N}{\text{O}}_{3}^{-}$ calibration showed a linear relationship of 53 mV/pNO_3_.

For measurements, the microelectrode voltage barrel was backfilled with 0.5 M KCl (Fernández et al., 1999; Rubio et al., 2005). Then, the $\text{N}{\text{O}}_{3}^{-}$- or Cl^−^ *-*selective microelectrode and the reference electrode (containing agar 0.03% (w/v) in 0.5 M KCl) were fixed to Ag/AgCl electrode holders and connected to a high-impedance differential amplifier (FD223a, WPI, Sarasota, Florida, USA). Amplifier signals were continuously monitored on a double pen chart recorder (Linseis L250E).

Measurements were performed on leaf pieces (≈ 1 cm long), longitudinally peeled to remove part of the epidermis and fixed with paraffin wax on a Plexiglas transparent chamber (1.1 ml volume). A gravity-based flow-through system permitted controlled changes of the assay medium at a rate of 10 ml·min^−1^, which renewed chamber volume approximately 10 times every minute. This system kept the temperature, the ionic concentration, and gases constant during the experiments. Measurements were made under a microscope light of 150 µmol photons·m^−2^·s^−1^.

### $\text{N}{\text{O}}_{3}^{-}$ Quantification in Assay Solutions

In order to monitor the net efflux and/or uptake of $\text{N}{\text{O}}_{3}^{-}$ from assay medium, plants were previously adapted to N-sufficiency by incubation in NSW enriched with 100 µM NaNO_3_ during 2 days. Then, excised leaves (2–3 g fresh weight) were placed separately in 250 ml flasks and incubated in 100 ml NSW (control) or NSW supplemented with 3 mM NaHCO_3_. The assay was carried out at 25°C with gentle and constant agitation. For each treatment, samples of assay medium were taken at 0, 1, 2, 3, 5, 7, 10, 15, 20, and 30 min. The highly sensitive method for $\text{N}{\text{O}}_{3}^{-}$ determination, based on vanadate $\text{N}{\text{O}}_{3}^{-}$reduction and the subsequent spectrophotometric determination of NO_2_^−^ (García-Robledo et al., 2014) was used to quantify $\text{N}{\text{O}}_{3}^{-}$ concentration in each sample. Net efflux and uptake rates were estimated as the slope of the linear phase of $\text{N}{\text{O}}_{3}^{-}$-concentration time course, as a function of fresh weight (FW). Six replicates were conducted for each assay.

### Assay Solutions

Natural seawater (NSW) supplemented with 3 mM $\text{HC}{\text{O}}_{3}^{-}$ was prepared by adding the appropriate volume of a 0.5 M NaHCO_3_ stock solution (pH 8.2). CO_2_-enriched NSW was obtained by bubbling with artificial air (Air Liquide, Spain, 1000 ppm CO_2_-air). A stock solution of the plasma membrane-permeable carbonic anhydrase inhibitor ethoxyzolamide (EZ, 10 mM) was prepared in 0.05 M NaOH. The addition of equivalent volumes of NaOH without the inhibitor had no effect on measurements. All chemicals were analytical grade and were purchased from Sigma-Aldrich.

### Data Presentation and Statistical Analyses

Time-course measurements are shown as single traces, representative of a number of equivalent experiments carried out under the same conditions, as stated in the figure legends. Data are presented as means, and error bars are standard deviations. Number of repetitions (n) is indicated in every experiment. Data were analyzed using SPSS Statistics, version 21. The significance level was set at *P* < 0.05.

## Results

### Effect of Light-Dark Transitions on *P. oceanica* Cytosolic $\text{N}{\text{O}}_{3}^{-}$

**Figure 1:**
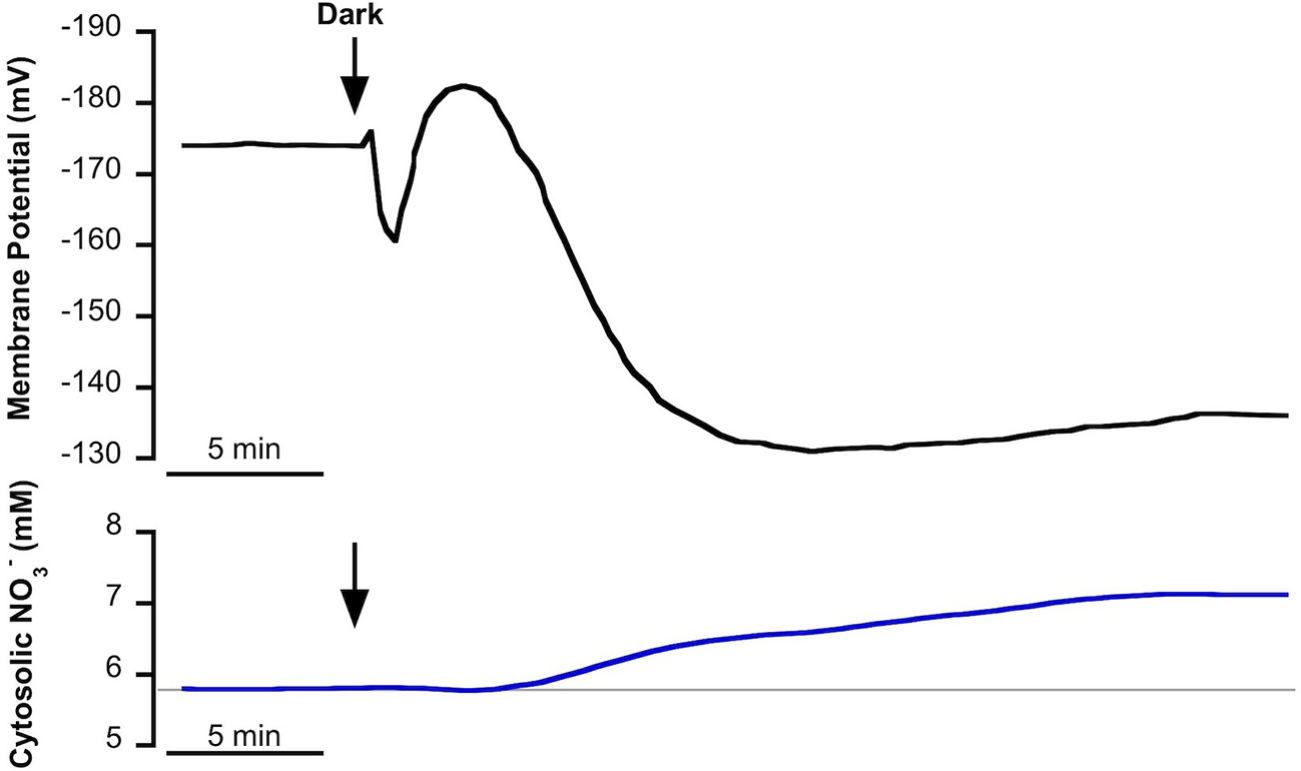
Effect of light-dark transition on plasma membrane potential (*Em*, mV) and cytosolic $\text{N}{\text{O}}_{3}^{-}$ (mM) in *Posidonia oceanica* mesophyll leaf cells, incubated in natural seawater. Traces are representative examples of intracellular nitrate-selective microelectrode recordings from 10 independent experiments. Arrows indicate the onset of dark treatment. Auxiliary grey line shows the standard cytosolic $\text{N}{\text{O}}_{3}^{-}$ concentration under light conditions (150 µmol photons m^−2^ s^−1^). Mean values and statistics are indicated in the text.

In NSW, mesophyll leaf cells of *P. oceanica* showed a stable plasma membrane potential of −174 ± 8 mV (as in Rubio et al., 2017) and a cytosolic $\text{N}{\text{O}}_{3}^{-}$ concentration of 5.7 ± 0.2 mM (n = 10). The transition from light to darkness evoked a fast depolarization of approximately 14 mV followed by a transient hyperpolarization of 22 mV and a subsequent, more prolonged, depolarization to level of at a lower membrane potential of −140 ± 5 mV (n = 12, *P* = 0.003, Student *t* test) after 25 min in the dark. With a delay of a few minutes, light-dark transition promoted the gradual increase of cytosolic $\text{N}{\text{O}}_{3}^{-}$ concentration that stabilized at a higher value (7.2 ± 0.6 mM $\text{N}{\text{O}}_{3}^{-}$; n = 10, *P* = 0.02, Student *t* test) after 30 min in the dark (**Figure 1**).

### Effect of $\text{HC}{\text{O}}_{3}^{-}$-Enriched NSW on Cytosolic $\text{N}{\text{O}}_{3}^{-}$

**Figure 2:**
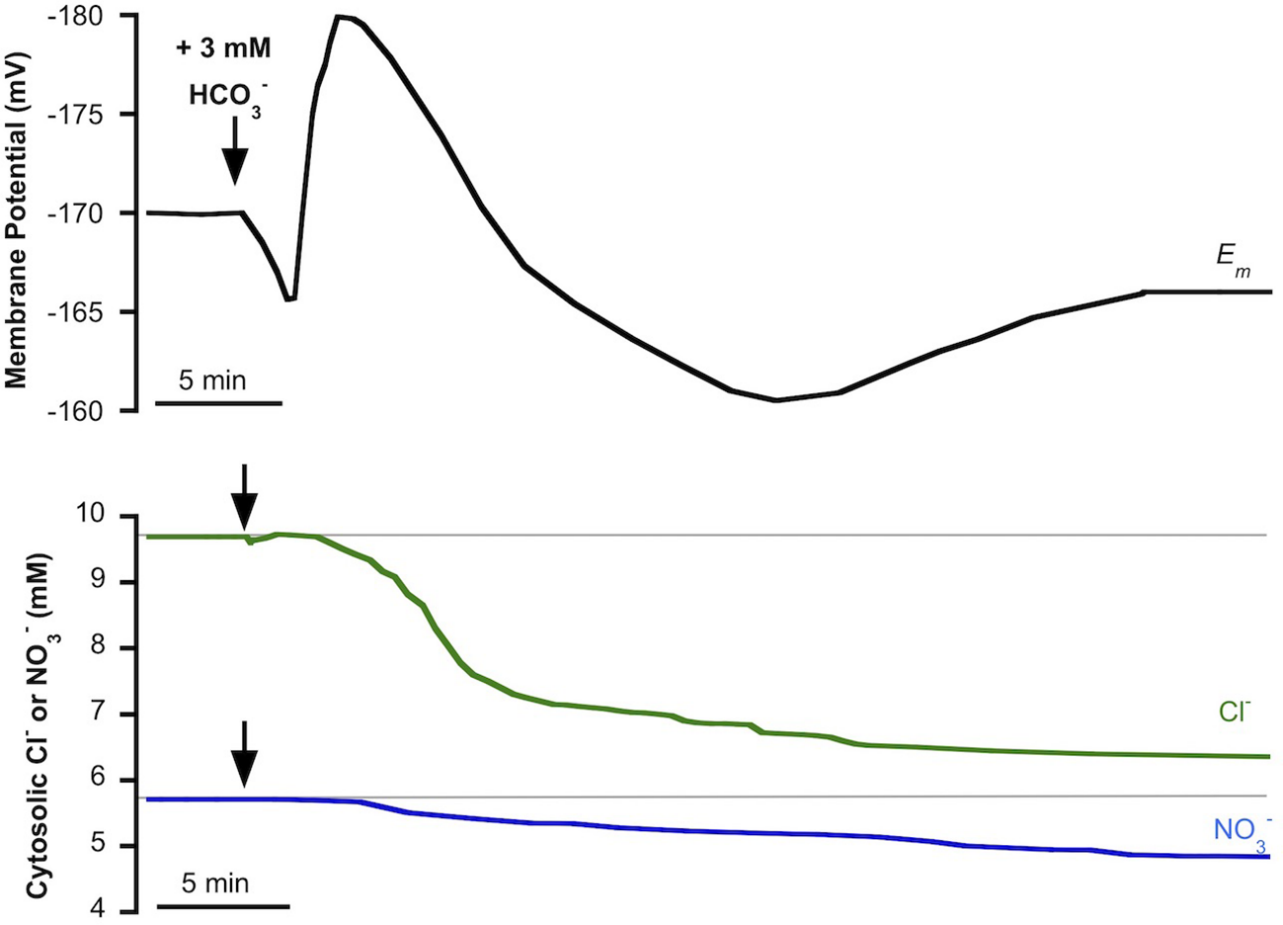
Effect of the addition of 3 mM $\text{HC}{\text{O}}_{3}^{-}$ on the plasma membrane potential (*Em*, mV), cytosolic chloride (Cl^−^, mM) or cytosolic $\text{N}{\text{O}}_{3}^{-}$ ($\text{N}{\text{O}}_{3}^{-}$, mM) measured in mesophyll leaf cells of *Posidonia oceanica*. Assay medium consisted of natural seawater and arrows indicate the addition of 3 mM $\text{HC}{\text{O}}_{3}^{-}$. Traces are representative examples from five independent recordings using intracellular Cl^−^-selective (green trace) or $\text{N}{\text{O}}_{3}^{-}$-selective (blue trace) microelectrodes, respectively. Auxiliary grey lines indicate the normal cytosolic Cl^−^ or $\text{N}{\text{O}}_{3}^{-}$ concentrations before $\text{HC}{\text{O}}_{3}^{-}$ addition. Mean values and statistics are indicated in the text and in **Table 1**.

We have previously reported that in *P. oceanica* mesophyll leaf cells, incubated in light conditions, the addition of NSW enriched with 3 mM $\text{HC}{\text{O}}_{3}^{-}$ evoked an initial and transient plasma membrane depolarization that turned into a transient hyperpolarization to stabilize at a depolarized value (Rubio et al., 2017). The simultaneous measurement of cytosolic chloride showed a delayed but significant decrease of the cytosolic concentration of this anion concomitant with the extent of membrane depolarization (Rubio et al., 2017). The partial sensitivity of Cl^−^-selective microelectrodes to $\text{N}{\text{O}}_{3}^{-}$ (Miller and Zhen, 1991; Felle, 1994) allows us to hypothesize that $\text{HC}{\text{O}}_{3}^{-}$ enrichment not only produces the cytosolic Cl^−^ decrease but could also evoke a cytosolic $\text{N}{\text{O}}_{3}^{-}$ shift. To test this hypothesis, cytosolic $\text{N}{\text{O}}_{3}^{-}$ was measured in *P. oceanica* mesophyll leaf cells in the same conditions that we had reported for cytosolic chloride, the monitoring of which was used as a control in this work. **Figure 2** shows the membrane potential response to the enrichment of NSW with 3 mM $\text{HC}{\text{O}}_{3}^{-}$ (black trace) and the simultaneous measurements of cytosolic Cl^−^ (green trace) or $\text{N}{\text{O}}_{3}^{-}$ (blue trace), values and stats are presented in **Table 1**. As found previously (Rubio et al., 2017), the addition of 3 mM $\text{HC}{\text{O}}_{3}^{-}$ caused an initial and transient membrane depolarization (black trace) of approximately 5 mV, which turned into a transient hyperpolarization (reaching a minimum membrane potential of −181 ± 2 mV, n = 5, *P* = 0.012, Student *t* test), followed by a prolonged depolarization that stabilized within approximately 40 min at a membrane potential of −168 ± 3 (n = 5). Also in agreement with the previous study, the addition of 3 mM $\text{HC}{\text{O}}_{3}^{-}$ evoked a decrease of cytosolic Cl^−^ (after approximately 4 min) which continued progressively from 9.7 ± 0.2 mM to 6.3 ± 0.3 mM (green trace, n = 5; *P* = 0.004, Student *t* test) 20 min after $\text{HC}{\text{O}}_{3}^{-}$ addition. Supporting the hypothesis of $\text{HC}{\text{O}}_{3}^{-}$ enrichment's producing a cytosolic $\text{N}{\text{O}}_{3}^{-}$ shift, *P. oceanica* mesophyll leaf cell cytosolic $\text{N}{\text{O}}_{3}^{-}$ also decreased in response to the enrichment of NSW with 3 mM $\text{HC}{\text{O}}_{3}^{-}$ (**Figure 2**, blue trace). In common with the response of cytosolic Cl^−^, the diminution of cytosolic $\text{N}{\text{O}}_{3}^{-}$ started approximately 4 min after $\text{HC}{\text{O}}_{3}^{-}$ treatment. Cytosolic $\text{N}{\text{O}}_{3}^{-}$ diminished gradually from 5.7 ± 0.2 mM to a steady lower value of 4.7 ± 0.1 mM after 35 min of $\text{HC}{\text{O}}_{3}^{-}$ addition, resulting in a significant cytosolic $\text{N}{\text{O}}_{3}^{-}$ shift of 0.9 ± 0.06 mM (n = 5; *P* = 0.03, Student *t* test). In both cases, time courses of cytosolic $\text{N}{\text{O}}_{3}^{-}$ and cytosolic Cl^−^ decreases aligned with the recovery of the transient membrane hyperpolarization and profound membrane depolarization, supporting a cytosolic leak of negative charge induced by the $\text{HC}{\text{O}}_{3}^{-}$ addition.

**Table 1:** Membrane potential (mV), cytosolic $\text{N}{\text{O}}_{3}^{-}$ (mM), and cytosolic Cl^−^ (mM) measured in mesophyll leaf cells of *P. oceanica* incubated in natural seawater (NSW) supplemented with different inorganic carbon concentrations.

	Membrane Potential (mV)	Cytosolic $\text{N}{\text{O}}_{3}^{-}$ (mM)	Cytosolic Cl^−^ (mM)
NSW	−170 ± 8 initial value	5.7 ± 0.2 initial concentration	9.7 ± 0.2 initial concentration
+3 mM $\text{HC}{\text{O}}_{3}^{-}$	−181 ± 2* initial hyperpolarization −168 ± 3 final value	4.7 ± 0.1* final concentration	6.3 ± 0.3* final concentration
+1000 ppmCO_2_	−174 ± 3 initial hyperpolarization −165 ± 3 final value	5.3 ± 0.6 final concentration	
+1000 ppmCO_2_ (0.1 mM EZ)	−178 ± 2 final value	5.6 ± 0.1 final concentration	

EZ is the carbonic anhydrase inhibitor, ethoxyzolamide. Values are mean ± SD of a number of experiments indicated in the text. Asterisks (*) denote significant differences with respect to control conditions (NSW), the statistical values are indicated in the text.

### Effect of CO_2_ Increase on Cytosolic $\text{N}{\text{O}}_{3}^{-}$

**Figure 3:**
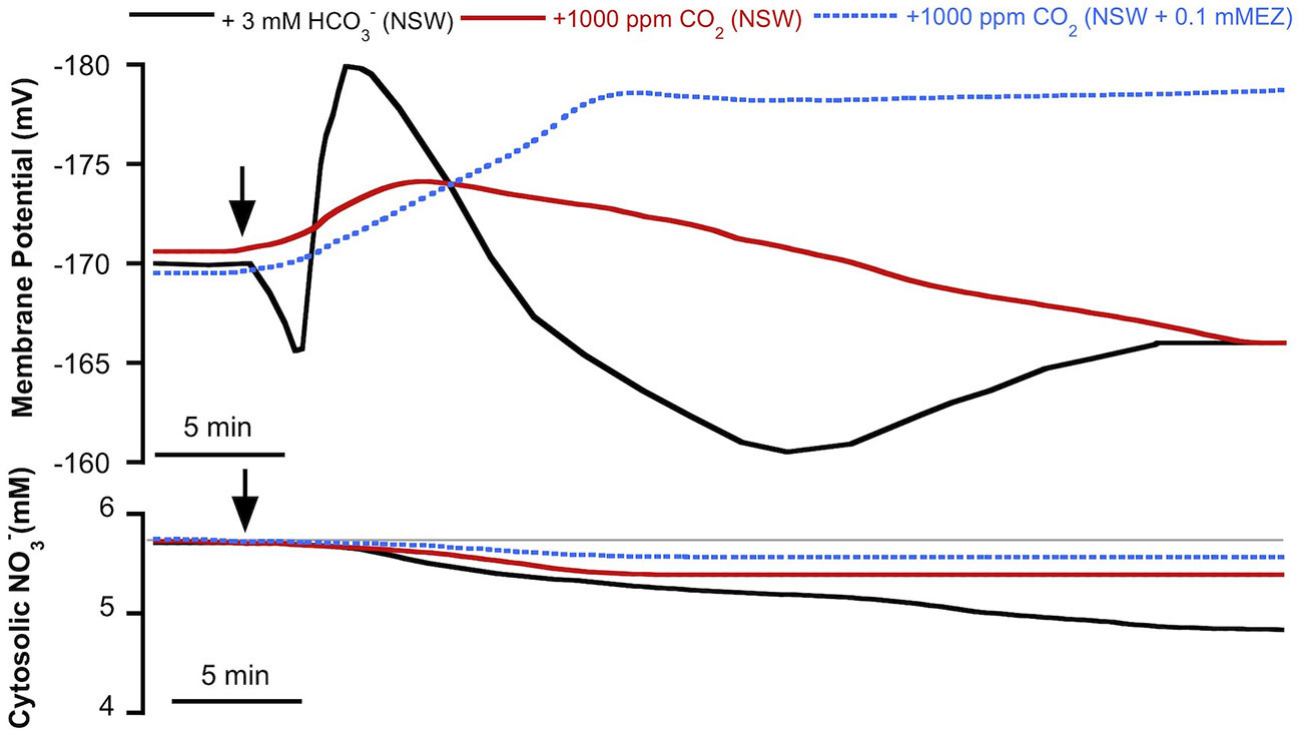
Effect of inorganic carbon increase on the plasma membrane potential (*Em*, mV) and cytosolic $\text{N}{\text{O}}_{3}^{-}$ (mM) measured in *Posidonia oceanica* mesophyll leaf cells. Arrows indicate the onset of 3 mM $\text{HC}{\text{O}}_{3}^{-}$ addition to natural seawater (NSW, black traces, control conditions) and the addition of 1,000 ppm CO_2_ to NSW (red traces) or to NSW containing 0.1 mM ethoxyzolamide (EZ, dashed blue traces). Traces are representative records of a minimum of four independent experiments using intracellular $\text{N}{\text{O}}_{3}^{-}$-selective microelectrodes. Mean values and statistics are indicated in the text and **Table 1**. Auxiliary grey line represents the normal cytosolic $\text{N}{\text{O}}_{3}^{-}$ concentration before the inorganic carbon additions.

As we have previously reported, in *P. oceanica* mesophyll leaf cells the direct uptake of $\text{HC}{\text{O}}_{3}^{-}$ and the subsequent use of CO_2_ for photosynthesis has consequences for anion homeostasis (Rubio et al., 2017). In this work, we show that $\text{HC}{\text{O}}_{3}^{-}$ use in this plant has an effect not only on cytosolic Cl^−^ but also on cytosolic $\text{N}{\text{O}}_{3}^{-}$ (**Figure 2**). The match of membrane depolarization and the onset of cytosolic Cl^−^ and $\text{N}{\text{O}}_{3}^{-}$ diminution suggests the involvement of plasma membrane S-type anion channels, which are permeable to both anions and whose activation takes place when cells depolarize (Schmidt et al., 1995; Roelfsema et al., 2004; Roberts, 2006). In terrestrial angiosperm guard cells, these channels are activated when the concentration of $\text{HC}{\text{O}}_{3}^{-}$ (not CO_2_) increases and the cytosolic pH (pHc) is alkaline (Xue et al., 2011). These are similar conditions to those observed in *P. oceanica* in the presence of high $\text{HC}{\text{O}}_{3}^{-}$ (Rubio et al., 2017). In order to rule out the role of CO_2_, cytosolic $\text{N}{\text{O}}_{3}^{-}$ was measured in mesophyll leaf cells of *P. oceanica* incubated in NSW supplemented with 1000 ppm CO_2_, in the absence or in the presence of the plasma membrane-permeable carbon anhydrase inhibitor ethoxyzolamide (0.1 mM EZ; Sültemeyer et al., 1993). As shown in **Figure 3** (data are summarized in **Table 1**), the increase of CO_2_ in NSW evoked a gradual plasma membrane hyperpolarization, reaching a value of −174 ± 3 mV (n = 5) after 7 min of 1000 ppm CO_2_ treatment. This maximum hyperpolarization was lower and almost 2 min delayed compared to that induced by the treatment with 3 mM $\text{HC}{\text{O}}_{3}^{-}$, included in **Figure 3** as control (black trace). Cytosolic $\text{N}{\text{O}}_{3}^{-}$ also decreased after the addition 1000 ppm CO_2_; however, in these conditions cytosolic $\text{N}{\text{O}}_{3}^{-}$ diminution was lower than that induced by 3 mM $\text{HC}{\text{O}}_{3}^{-}$ treatment. After 15 min in the presence of 1000 ppm CO_2_, cytosolic $\text{N}{\text{O}}_{3}^{-}$ stabilized at a concentration of 5.3 ± 0.6 mM, a statistically non significant shift (n = 5; *P =* 0.37, Student *t* test). Furthermore, in the presence of 0.1 mM EZ, the enrichment of NSW with 1000 ppm CO_2_ evoked a marked plasma membrane hyperpolarization that reached a steady maximum value of −178 ± 2 mV after 12 min treatment. In the presence of EZ, cytosolic $\text{N}{\text{O}}_{3}^{-}$ showed a minimum, statistically non-significant, shift from 5.7 ± 0.2 to 5.6 ± 0.1 mM $\text{N}{\text{O}}_{3}^{-}$ (n = 4; *P* = 0.53, Student *t* test). That neither the long-term depolarization nor the drop in cytosolic $\text{N}{\text{O}}_{3}^{-}$ content evoked by $\text{HC}{\text{O}}_{3}^{-}$ were evident with CO_2_ in the presence of EZ (thus restricting $\text{HC}{\text{O}}_{3}^{-}$ production from the CO_2_ source) points to the need for substantial cytosolic $\text{HC}{\text{O}}_{3}^{-}$ accumulation to effect ionic fluxes. Accordingly, these results strongly indicate that the cytosolic $\text{N}{\text{O}}_{3}^{-}$ decrease is caused by the $\text{HC}{\text{O}}_{3}^{-}$ enrichment and point to the S-type anion channel activation by the cytosolic $\text{HC}{\text{O}}_{3}^{-}$ (not CO_2_) increase.

### Effect of $\text{HC}{\text{O}}_{3}^{-}$ Increase on External $\text{N}{\text{O}}_{3}^{-}$

**Figure 4:**
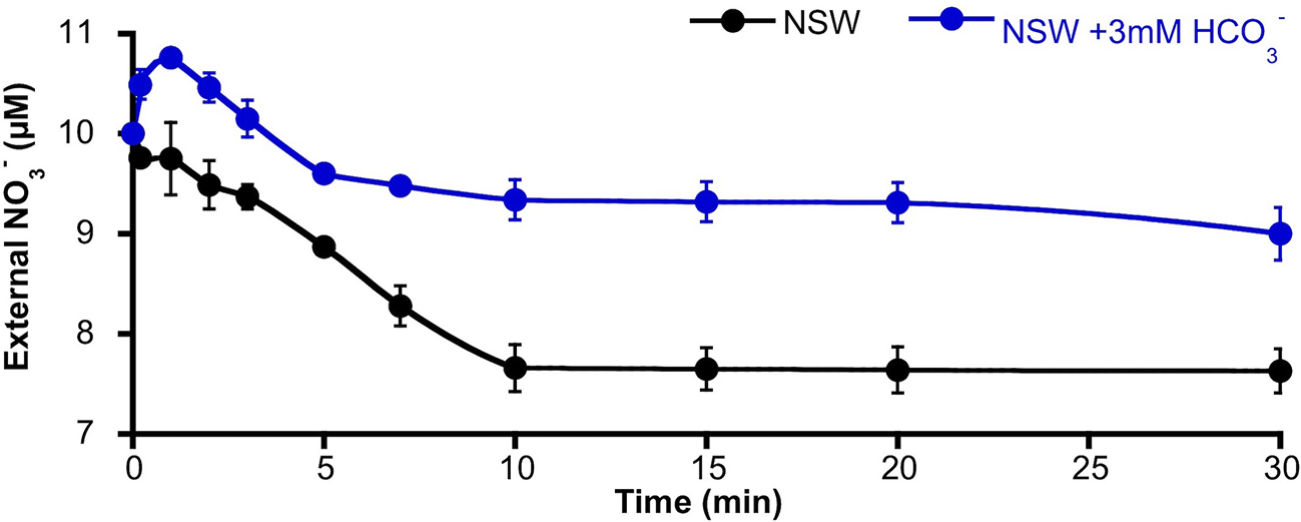
Time course of the external nitrate concentration around *Posidonia oceanica* leaf pieces incubated in NSW. Plants were previously incubated in NSW containing 100 µM $\text{N}{\text{O}}_{3}^{-}$, then excised leaves were incubated in NSW (black trace, control condition) or NSW supplemented with 3 mM $\text{HC}{\text{O}}_{3}^{-}$ (blue trace). At different times within the first 30 min of incubation, samples were taken and used to determinate external $\text{N}{\text{O}}_{3}^{-}$ concentration. Data are mean ± SD of six independent assays. Mean values, net efflux, and influx $\text{N}{\text{O}}_{3}^{-}$ rates and statistics are indicated in the text.

The activation of S-type anion channels may allow the efflux of $\text{N}{\text{O}}_{3}^{-}$ from mesophyll leaf cells; such a phenomenon of $\text{N}{\text{O}}_{3}^{-}$ efflux should be higher in the case of $\text{N}{\text{O}}_{3}^{-}$-replete cells. Thus, in order to investigate if the $\text{HC}{\text{O}}_{3}^{-}$ enrichment of NSW promoted $\text{N}{\text{O}}_{3}^{-}$ efflux from *P. oceanica* leaves, the time course of external $\text{N}{\text{O}}_{3}^{-}$ concentration change was monitored in assay medium containing leaves from plants pre-incubated for 2 days in NSW containing 100 µM $\text{N}{\text{O}}_{3}^{-}$. $\text{N}{\text{O}}_{3}^{-}$-supplied leaves were then incubated in NSW containing the standard $\text{N}{\text{O}}_{3}^{-}$ concentration (10 µM). After the addition of 3 mM $\text{HC}{\text{O}}_{3}^{-}$, external $\text{N}{\text{O}}_{3}^{-}$ concentration increased significantly (10.8 ± 0.04 µM $\text{N}{\text{O}}_{3}^{-}$; n = 6; *P <*0.001, Student *t* test) within the first minute of incubation. Maximum net efflux rate was 18 ± 2 nmol $\text{N}{\text{O}}_{3}^{-}$·g_FW_^−1^·min^−1^. Then, external $\text{N}{\text{O}}_{3}^{-}$ decreased at a net rate of 8 ± 1 nmol $\text{N}{\text{O}}_{3}^{-}$·g_FW_^−1^·min^−1^ until 5 min of incubation to stabilize at a concentration of 9.2 ± 0.1 µM $\text{N}{\text{O}}_{3}^{-}$ (**Figure 4**). Under control conditions (no $\text{HC}{\text{O}}_{3}^{-}$ addition), no external $\text{N}{\text{O}}_{3}^{-}$ increase was detected, but $\text{N}{\text{O}}_{3}^{-}$ concentration progressively decreased at a net rate of 11 ± 2 nmol $\text{N}{\text{O}}_{3}^{-}$·g_FW_^−1^·min^−1^ during the first 10 min of incubation to reach a steady lower value of 7.6 ± 0.2 µM $\text{N}{\text{O}}_{3}^{-}$ (n = 6; *P* = 0.002, Student *t* test). External $\text{N}{\text{O}}_{3}^{-}$ concentrations showed higher values in all samples from leaves incubated in NSW enriched with 3 mM $\text{HC}{\text{O}}_{3}^{-}$ suggesting a net $\text{N}{\text{O}}_{3}^{-}$ efflux from *P. oceanica* leaves under these conditions.

## Discussion

### Cytosolic $\text{N}{\text{O}}_{3}^{-}$ Changes During Light–Dark Transitions in *P. oceanica* Leaf Cells

The concentration of $\text{N}{\text{O}}_{3}^{-}$ in the cytosol ($\text{N}{\text{O}}_{3}^{-}{}_{\text{c}}$) depends on a series of highly regulated processes at the cellular level that includes sensing and transport at the plasma membrane (uptake and efflux), subcellular compartmentalization and metabolism. Depending on the technique and the plant system used, a wide range of cytosolic $\text{N}{\text{O}}_{3}^{-}$ values has been reported with also a large degree of variability. Therefore some authors suggest that $\text{N}{\text{O}}_{3}^{-}{}_{\text{c}}$ would not be subject to homeostasis, as would be the case for cytosolic pH or Ca^2+^ for example (for discussion see Siddiqi and Glass, 2002 or Miller and Smith, 2008). After the development of intracellular $\text{N}{\text{O}}_{3}^{-}$-selective microelectrodes by Miller and Zhen (1991) it is possible to perform continuous measurements of the cytosolic free ion activity of $\text{N}{\text{O}}_{3}^{-}$ with the possibility to obtain instantaneous responses to changes in the experimental conditions. This approach has been used here to report the first cytosolic $\text{N}{\text{O}}_{3}^{-}$ values of a marine vascular plant.

Under light conditions the cytosolic $\text{N}{\text{O}}_{3}^{-}$ measured using intracellular $\text{N}{\text{O}}_{3}^{-}$-selective microelectrodes in *P. oceanica* mesophyll leaf cells was 5.7 ± 0.2 mM (n = 10), almost double the value reported for both epidermal and mesophyll leaf cells of *A. thaliana* (2.2 and 2.8 mM $\text{N}{\text{O}}_{3}^{-}{}_{\text{c}}$, respectively; Cookson et al., 2005). This cytosolic $\text{N}{\text{O}}_{3}^{-}$ in mesophyll leaf cells of *P. oceanica* is also much higher than that reported for the intermodal cells of the freshwater alga *C. corallina* (1.6 mM $\text{N}{\text{O}}_{3}^{-}$; Miller and Zhen, 1991) or in the liverwort *C. conicum* (0.63 mM $\text{N}{\text{O}}_{3}^{-}$; Trębacz et al., 1994), using the same technique. However, the $\text{N}{\text{O}}_{3}^{-}{}_{\text{c}}$ value found in *P. oceanica* is similar to those observed in root cells of barley (5.4 mM $\text{N}{\text{O}}_{3}^{-}$; Zhen et al., 1991) or maize (3.1 mM $\text{N}{\text{O}}_{3}^{-}$; Miller and Smith, 1996).

The $\text{N}{\text{O}}_{3}^{-}{}_{\text{c}}$ value in mesophyll leaf cells of *P. oceanica* is lower than the cytosolic Cl^−^ concentration (Cl^−^c) of 9.7 mM Cl^−^, yielding a $\text{N}{\text{O}}_{3}^{-}{}_{\text{c}}$/Cl^−^c of 0.6. This ratio is higher than that calculated for *C. conicum,* $\text{N}{\text{O}}_{3}^{-}{}_{\text{c}}$/Cl^−^c ≈ 0.1, taking 7.4 mM as the Cl^−^c (Trębacz et al., 1994), but it is in the range of the estimates for root cells of barley ($\text{N}{\text{O}}_{3}^{-}{}_{\text{c}}$/Cl^−^c ≈ 0.9) or *A. thaliana* mesophyll leaf cells ($\text{N}{\text{O}}_{3}^{-}{}_{\text{c}}$/Cl^−^c ≈ 0.3). Those values have been calculated considering Cl^−^c as 6 mM for barley root cells (Britto et al., 2004) and assuming that the reported cytosolic Cl^−^ in *A. thaliana* root cells (8.7 mM Cl^−^c; Planes et al., 2015) would be the same in mesophyll leaf cells.

Changes in environmental conditions have been related to $\text{N}{\text{O}}_{3}^{-}{}_{\text{c}}$ alterations, supporting the idea that the cytosol operates a strong ion homeostasis not only for pH, Pi or Ca^2+^, but also for $\text{N}{\text{O}}_{3}^{-}$ (reviewed by Miller and Smith, 2008). Light-dark transition triggers a slight increase of $\text{N}{\text{O}}_{3}^{-}{}_{\text{c}}$ in the liverwort *C. conicum* (Trębacz et al., 1994) and a transient increase, from 2 to 3.5 mM, which peaked after 7 min of darkness in the case of *A. thaliana* leaf cells (Cookson et al., 2005). A similar response has been found here in *P. oceanica* mesophyll leaf cells, in which cytosolic $\text{N}{\text{O}}_{3}^{-}$ reached a maximum value of 7.2 mM after 25 min of dark treatment. In the case of *A. thaliana*, since the effect was not observed when measurements were performed in the nitrate reductase (NR) mutant (*nia1nia2*) leaf cells, the $\text{N}{\text{O}}_{3}^{-}{}_{\text{c}}$ increase was explained because the shift to the dark inactivates NR, leading to a transient build-up of $\text{N}{\text{O}}_{3}^{-}{}_{\text{c}}$ due to a slower reduction rate (Cookson et al., 2005). A higher $\text{N}{\text{O}}_{3}^{-}{}_{\text{c}}$ in *A. thaliana nia1nia2* mesophyll leaf cells and a similar time course of cytosolic increase to the rate of NR activity change in response to illumination transitions (half-life of 2 to 15 min in spinach; Huber et al., 1992; Kaiser et al., 1992; Riens and Heldt, 1992) support the evidence for the role of NR in regulating $\text{N}{\text{O}}_{3}^{-}{}_{\text{c}}$ (Cookson et al., 2005). In *P. oceanica*, the $\text{N}{\text{O}}_{3}^{-}{}_{\text{c}}$ increase observed in the dark could also explain the one-half diminution of the maximum high affinity $\text{N}{\text{O}}_{3}^{-}$ uptake observed previously in mesophyll leaf cells of this plant (Rubio et al., 2018), due to the apparent substrate inhibition of the transporter.

### Inorganic Carbon Increase Triggers Cytosolic $\text{N}{\text{O}}_{3}^{-}$ Decrease

In a previous work we demonstrated that a plasma membrane *n*H^+^/$\text{HC}{\text{O}}_{3}^{-}$ symporter mediates the uptake of $\text{HC}{\text{O}}_{3}^{-}$ in *P. oceanica* mesophyll leaf cells. Further, the direct uptake of $\text{HC}{\text{O}}_{3}^{-}$ followed by its internal dehydration renders CO_2_ (used for photosynthesis) and hydroxyl anions, promoting membrane potential and cytosolic pH and Cl^−^ variations (Rubio et al., 2017). In the present work, we have also verified that uptake of $\text{HC}{\text{O}}_{3}^{-}$ promotes the decrease of $\text{N}{\text{O}}_{3}^{-}{}_{\text{c}}$ in *P. oceanica* mesophyll leaf cells. The increases of cytosolic $\text{HC}{\text{O}}_{3}^{-}$ and cytosolic pH have been related to promoting the opening of plasma membrane S-type anion channels in guard cells (Xue et al., 2011). A similar pathway seems to explain the $\text{N}{\text{O}}_{3}^{-}{}_{\text{c}}$ diminution observed in response to $\text{HC}{\text{O}}_{3}^{-}$ addition in *P. oceanica* mesophyll leaf cells, as was previously proposed in the case of Cl^−^c (Rubio et al., 2017), used as a control in this work. In both cases, the onset of $\text{N}{\text{O}}_{3}^{-}{}_{\text{c}}$ and Cl^−^c decreases matched that of the plasma membrane depolarization, and indeed depolarization is a required initial phase to activate S-type anion channels operating at the plasma membrane of guard cells (Schmidt et al., 1995; Roelfsema et al., 2004; Roberts, 2006).

S-type anion channels are encoded by the small *SLAC/SLAH* gene family, that share homology to transport systems found in different kingdoms (Dreyer et al., 2012). In *A. thaliana*, apart from *SLAC1* that is exclusively expressed in guard cells, four additional homologs (*SLAH1-4*) are present (Negi et al., 2008). SLAC1 and SLAH3 channels exhibit a permeability preference for $\text{N}{\text{O}}_{3}^{-}$ and Cl^−^ but not for malate (Schmidt and Schroeder, 1994; Geiger et al., 2009; Chen et al., 2010). The SLAH3 channel has the highest permeability for $\text{N}{\text{O}}_{3}^{-}$ ($\text{N}{\text{O}}_{3}^{-}$/Cl^−^ permeability ratio of 20) and in contrast to SLAC1, SLAH3 also requires extracellular $\text{N}{\text{O}}_{3}^{-}$ to induce its activity (Geiger et al., 2011). Furthermore, a role in $\text{N}{\text{O}}_{3}^{-}$-dependent alleviation of ammonium toxicity in *A. thaliana* roots has been proposed for SLAH3 (Zheng et al., 2015).

Although seagrasses have lost stomata differentiation genes (Olsen et al., 2016) the presence of S-type anion channels cannot be ruled out in those plants since, presumably, these anion channels have evolved as emergency valves, rapidly releasing excess osmolytes under stress conditions (Roelfsema et al., 2012). The *Zostera marina* genome, the first one available from a seagrass (Olsen et al., 2016), contains two homologs for the *A. thaliana SLAC/SLAH* gene family (*Phytozome v12.1*, data base), one *SLAH1* homolog (*Zosma91g00860.1*) and one *SLAC1* homolog (*Zosma76g00610.1*). The presumed homologs of these channels in *P. oceanica* should be good candidates for the proposed channels for the leak of $\text{N}{\text{O}}_{3}^{-}$ and Cl^−^ ions from the cytosol, but further molecular analyses are needed to characterize the specific role of these anion channels to mediate anion efflux in response to high $\text{HC}{\text{O}}_{3}^{-}$.

**Figure 5:**
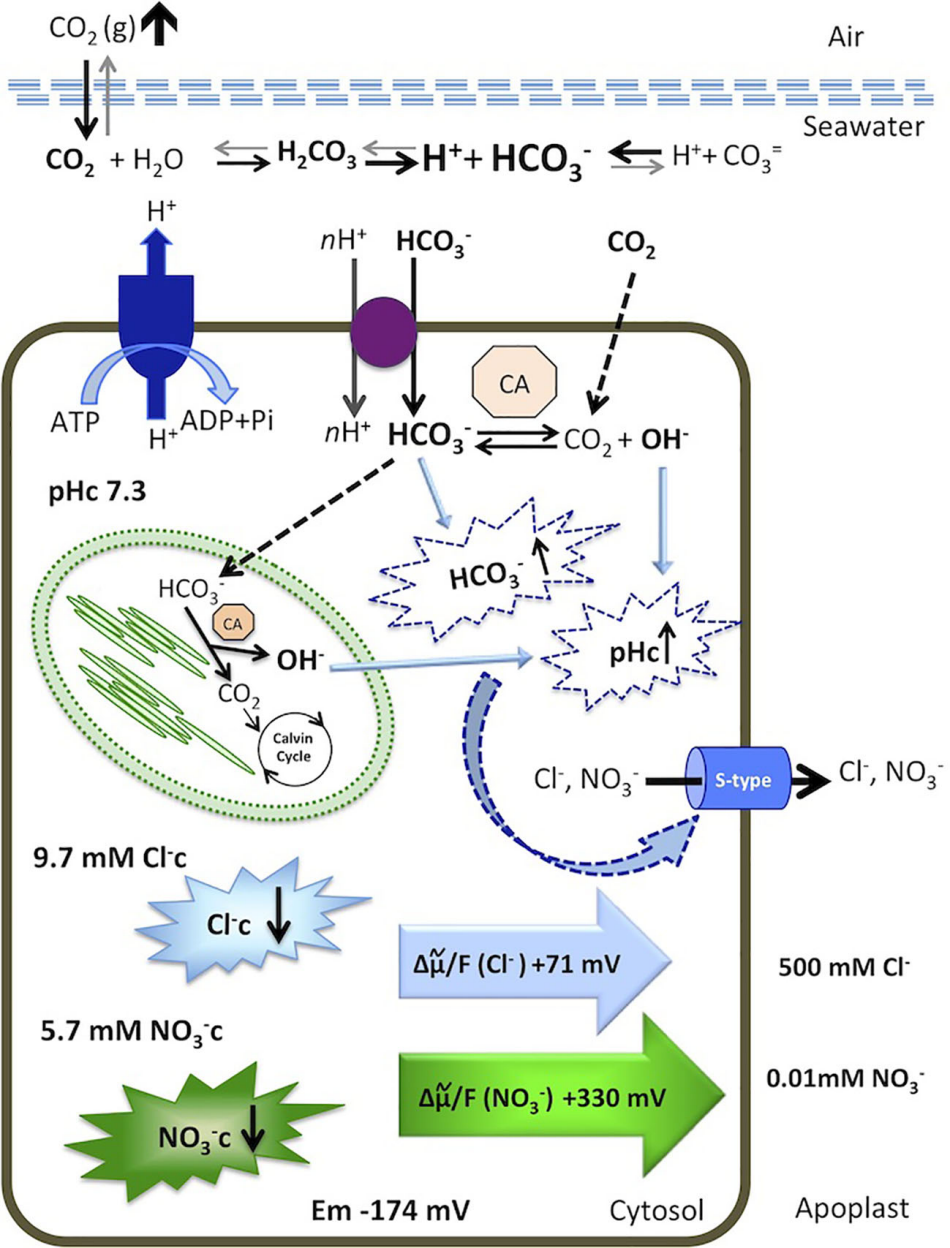
Model for cytosolic $\text{N}{\text{O}}_{3}^{-}$ and Cl^−^ responses to an increase of dissolved inorganic carbon in the marine angiosperm *Posidonia oceanica* leaf cells. Elevated atmospheric CO_2_ level increases total dissolved inorganic carbon, shifting the equilibrium in favor of $\text{HC}{\text{O}}_{3}^{-}$ in the seawater. The $\text{HC}{\text{O}}_{3}^{-}$ is taken up by an H^+^-symport and dehydrated in the cytosol or transported to the chloroplast serving as inorganic carbon source for photosynthesis. Internal dehydration, catalyzed by the carbonic anhydrases (CA) renders CO_2_, consumed by the Calvin Cycle, and OH^-^ ions that increase cytosolic pH (pHc). Excess of cytosolic $\text{HC}{\text{O}}_{3}^{-}$ and elevated pHc activates S-type anion channels, through which Cl^−^ and $\text{N}{\text{O}}_{3}^{-}$ leave the cells by an outwardly directed motive force of +71 mV and +330 mV, respectively. Consequently, the cytosolic concentrations of both anions decrease. Membrane potential, ion concentrations, calculations, and discussion are included in the text.

In guard cells, stomatal closure in response to high CO_2_ is mediated by the activation of S-type (SLAC1/SLAH3) anion channels (reviewed by Hedrich and Geiger, 2017). Interestingly, guard cells do not sense CO_2_ themselves, but instead HCO_3_^–^ synthesized from CO_2_ within the cytosol by carbonic anhydrases (Xue et al., 2011). The same case could be proposed in *P. oceanica* mesophyll leaf cells, since no significant $\text{N}{\text{O}}_{3}^{-}{}_{\text{c}}$ decrease was found in response to 1000 ppm CO_2_ treatment in either the absence or the presence of the plasma membrane-permeable carbon anhydrase inhibitor ethoxyzolamide, suggesting that $\text{HC}{\text{O}}_{3}^{-}$, not CO_2_, is the inorganic carbon species that triggers $\text{N}{\text{O}}_{3}^{-}{}_{\text{c}}$ efflux from *P. oceanica* mesophyll leaf cells (**Figure 5**). Furthermore, probably due to the activation of the H^+^-pump in response to the cytosolic accumulation of CO_2_, a weak acid, the 1000 ppm CO_2_ treatment renders the hyperpolarization of the plasma membrane and the cytosolic acidification (see Rubio et al., 2017). Both responses are prolonged in the presence of ethoxyzolamide, that impairs $\text{HC}{\text{O}}_{3}^{-}$ production from the CO_2_ source, leading to conditions that not support the activation of S-type anion channels described above and could explain the non-significant $\text{N}{\text{O}}_{3}^{-}{}_{\text{c}}$ diminution observed in response to CO_2_ increase.

### Cytosolic $\text{N}{\text{O}}_{3}^{-}$ Efflux Under High Inorganic Carbon Could Contribute to Biomass N-Impoverishment in Seagrasses

As we showed previously, direct $\text{HC}{\text{O}}_{3}^{-}$ uptake has consequences for cytosolic ion homeostasis in *P. oceanica* mesophyll leaf cells (Rubio et al., 2017). The short-term net efflux of $\text{N}{\text{O}}_{3}^{-}$ and the low $\text{N}{\text{O}}_{3}^{-}$ net uptake rate observed in the present work also indicate that natural seawater $\text{HC}{\text{O}}_{3}^{-}$ enrichment may be important not only for cytosolic ion homeostasis but for the metabolic flux of $\text{N}{\text{O}}_{3}^{-}$ in this seagrass. In natural seawater, containing 500 mM Cl^−^ and 10 µM $\text{N}{\text{O}}_{3}^{-}$, the efflux of Cl^−^ and $\text{N}{\text{O}}_{3}^{-}$ from *P. oceanica* mesophyll leaf cells is driven by the outwardly directed anion electrochemical potential gradients for both anions (**Figure 5**). This electrochemical potential gradient (in mV) is almost five-fold higher for $\text{N}{\text{O}}_{3}^{-}$ (+330 mV) than for Cl^−^ (+71 mV). However, in response to high $\text{HC}{\text{O}}_{3}^{-}$ the Cl^−^c decrease observed in *P. oceanica* mesophyll leaf cells (ΔCl^−^c = 3.4 mM) was much higher than for $\text{N}{\text{O}}_{3}^{-}{}_{\text{c}}$ (Δ$\text{N}{\text{O}}_{3}^{-}{}_{\text{c}}$ = 1 mM). This lower $\text{N}{\text{O}}_{3}^{-}{}_{\text{c}}$ leak than the expected from electrochemical potential gradient comparison with Cl^−^c could be explained by a different membrane permeability for both anions and/or different capability of compartmentalization. Nevertheless, even the observed decrease of $\text{N}{\text{O}}_{3}^{-}{}_{\text{c}}$, the short-term $\text{N}{\text{O}}_{3}^{-}$ efflux, and the low $\text{N}{\text{O}}_{3}^{-}$ uptake rate could impair N-assimilation in *P. oceanica* leaf cells in natural seawater containing high inorganic carbon.

An impaired metabolic flux of N should be relevant in the context of atmospheric CO_2_ rise. Oceans have been the sink for 30% of CO_2_ released during the industrial era, at a higher rate (3.8 GTons·year^−1^) than the 1.8 GTons·year^−1^ fixed by photosynthesis or the 2 GTons·year^−1^ removed by abiotic absorption (Behrenfeld et al., 2002). Atmospheric CO_2_ is exchanged into aquatic environments rendering the dissolved inorganic carbon (DIC) equilibrium. Controlled by pH, this equilibrium generates the distribution of DIC species: dissolved CO_2_, bicarbonate ($\text{HC}{\text{O}}_{3}^{-}$) and carbonate (CO_3_^2−^) ions. Consequently, elevated atmospheric CO_2_ concentration increases the total DIC and lowers the pH, shifting the relative proportion of each DIC species. Under current ocean pH (~8.04) and atmospheric CO_2_ (~ 410 ppm), the smallest pool of DIC is dissolved CO_2_, but this will have the greatest increase (> 250%) among the DIC constituents as the pH drops (~ 0.3–0.4 pH units) under the predicted rise in atmospheric CO_2_ (1000 ppm) for 2100. In contrast, the $\text{HC}{\text{O}}_{3}^{-}$ pool will only increase by 15% at that date (Koch et al., 2013). However, in terms of absolute concentration (mol·kg^−1^) $\text{HC}{\text{O}}_{3}^{-}$ levels will rise more than dissolved CO_2_ (Raven et al., 2005). Using the predicted data for 2100, calculations render 2.05 mmol·kg^−1^ $\text{HC}{\text{O}}_{3}^{-}$ and only 0.03 mmol·kg^−1^ CO_2_ (Koch et al., 2013). Considering such a $\text{HC}{\text{O}}_{3}^{-}$ rise (1.2 fold higher than actual concentration) and that the addition of 3 mM $\text{HC}{\text{O}}_{3}^{-}$ corresponds to an increase by 2.3 fold of the $\text{HC}{\text{O}}_{3}^{-}$ concentration in natural seawater, a $\text{N}{\text{O}}_{3}^{-}{}_{\text{c}}$ leak of 0.5 mM would be expected in *P. oceanica* mesophyll leaf cells by 2100. However, long-term effect of elevated atmospheric CO_2_ concentration on seagrasses N content needs further investigation, since N-deficiency induces high-affinity $\text{N}{\text{O}}_{3}^{-}$ uptake, which contributes to $\text{N}{\text{O}}_{3}^{-}$ homeostasis (reviewed by Rubio and Fernández, 2019).

Furthermore, the concomitant natural seawater pH decrease under elevated atmospheric CO_2_ could alter N availability in seawater and even the energy cost for nutrient uptake. A pH change from 8.1 to 7.8 evokes a decrease in the amount of NH_3_ in the NH_4_^+^/NH_3_ ratio (Raven, 1986), whereas the amount of $\text{N}{\text{O}}_{3}^{-}$ would not be affected (Zeebe and Wolf-Gladrow, 2001). Instead, the 0.3 external pH unit decrease renders a rise of the proton motive force at the plasma membrane of *P. oceanica* leaf cells of −18 mV, considering −174 mV as membrane potential, 7.3 as the cytosolic pH and constant temperature (Rubio et al., 2017). This amount represents, approximately, a 15% increase of the proton motive force (inwardly directed), which could prompt the activity of H^+^-dependent transport systems in this seagrass, including the plasma membrane H^+^/$\text{HC}{\text{O}}_{3}^{-}$ symporter (Rubio et al., 2017). As discussed by Rubio and Fernández (2019), ocean acidification could increase both the H^+^ and even the Na^+^ motive force due to changes in the activity of the plasma membrane Na^+^/H^+^ antiporter found in seagrass species (Rubio et al., 2011). The activity of this antiporter generates a lower cytosolic Na^+^ concentration at acid external pH and, consequently, a rise of the inwardly directed Na^+^ motive force could be also expected, favoring high-affinity $\text{N}{\text{O}}_{3}^{-}$ uptake based on Na^+^-dependent transport systems in seagrasses (reviewed by Rubio and Fernández, 2019). In fact, a recent study in *P. oceanica* beds from the Gulf of Naples (Italy) shows that the long-term exposure (9 months) to acidified seawater (pH 7.82) under constant DIC conditions promotes the diminution of the C/N molar ratio, due to the increase of N content by 21% and 70% in leaves and rhizome, respectively, whereas C content of those organs is not affected by external pH acidification (Scartazza et al., 2017). Those authors suggest that seawater acidification promoted a feed-forward long-term effect on N accumulation in *P. oceanica*, especially at the rhizome, although they recognize the need to specify the effect of acidification on the nutrient availability in their study (Scartazza et al., 2017). Interestingly, long-term nutrient enrichment seems to modulate the effects of ocean acidification on *P. oceanica*. Molecular analysis indicates that after 18 months at low pH (7.78) conditions the expression of nitrate transporter genes in *P. oceanica* leaves is altered; while *NRT1_6.3* and *NRT1_2.13* (involved in $\text{N}{\text{O}}_{3}^{-}$ sensing and low-affinity transport, respectively) are overexpressed the high-affinity $\text{N}{\text{O}}_{3}^{-}$ transporter gene *NRT2* shows a down-regulated expression (Ravaglioli et al., 2017). Thus, long-term overexpression of nitrogen transporter genes following nutrient additions at low pH suggests enhanced nutrient uptake and proposes that the effects of ocean acidification on *P. oceanica* depend upon local nutrient concentration (Ravaglioli et al., 2017).

Contrary to the acidification effect in *P. oceanica* meadows, several lines of evidence show that the most common effect of elevated CO_2_ is a decrease in the dry mass concentration of N in plant tissue (Cotrufo et al., 1998; Curtis and Wang, 1998; Norby et al., 1999; Jablonski et al., 2002; Ainsworth and Long, 2005; Taub et al., 2008). This suggests that physiological changes leading to decreased biomass N under elevated CO_2_ predominate in their effects over factors that would tend to increase N content (reviewed by Taub and Wang, 2008). Consequently, plants with non-saturated photosynthesis at actual atmospheric CO_2_, show an ionomic imbalance, with N the main nutrient that decreased at high C assimilation (Loladze, 2014). The physiological mechanisms responsible for this phenomenon have not been well established yet, although different hypotheses are proposed to account for it. In terrestrial vascular plants, the best-supported are the decrease of specific N uptake and assimilation due to a diminution of the transpiration-driven mass flow of N by a decreased stomatal conductance at elevated CO_2_ and the biomass dilution of N by increased photosynthetic assimilation of C (reviewed by Taub and Wang, 2008).

Seagrass meadows rank among the most productive ecosystems on Earth (Duarte and Chiscano, 1999), which largely contribute to C uptake in coastal waters and with most species capable of utilizing $\text{HC}{\text{O}}_{3}^{-}$ as a CO_2_ source for photosynthesis (Raven et al., 2014). With the exception of *Cymodocea nodosa* (considered C4), seagrasses show C3 photosynthetic pathways and are not saturated at the current ocean DIC concentration (reviewed by Koch et al., 2013). As occurs in C3 terrestrial plants, a higher C assimilation occurs in seagrasses due to the rise of DIC discussed above (Borum et al., 2016). The unpredicted effect of $\text{HC}{\text{O}}_{3}^{-}$ enrichment on anion homeostasis in *P. oceanica* mesophyll leaf cells, leading to the short-term efflux of $\text{N}{\text{O}}_{3}^{-}$ from the cytosol, possibly through the activation of S-type anion channels, supports a new mechanism as a key consideration in understanding the expected biomass N-alteration in seagrasses under elevated DIC, and probably, for terrestrial plants growing in waterlogged alkaline environments.

## Data Availability Statement

The datasets generated for this study are available on request to the corresponding author.

## Author Contributions

LR, DG-P, and JF performed the experiments. DG-P and LR accomplished data analysis. LR, JD, and JF discussed the results, wrote and edited the manuscript. LR and JF conceived the project. All authors contributed to the article and approved the submitted version.

## Funding

This work was supported by the Spanish Ministerio de Economía y Competitividad (cofinanced by the European Regional Development Fund; grant BFU2017-85117-R) awarded to JF and LR and the University of Cambridge's Central Chest (JD).

## Conflict of Interest

The authors declare that the research was conducted in the absence of any commercial or financial relationships that could be construed as a potential conflict of interest.

## References

[B1] Ainsworth E. A. Long S. P. (2005). What have we learned from 15 years of free-air CO_2_ enrichment (FACE)? A meta-analytic review of the responses of photosynthesis, canopy properties and plant production to rising CO_2_ . New Phytol. 165, 351– 372. 10.1111/j.1469-8137.2004.01224.x 15720649

[B2] Aires T. Marbà N. Cunha R. L. Kendrick G. A. Walker D. I. Serrão E. A. (2011). Evolutionary history of the seagrass genus Posidonia. Mar. Ecol. Prog. Ser. 421, 117–130. 10.3354/meps08879

[B3] Behrenfeld M. J. Esaias W. E. Turpie K. R. (2002). “Assessment of the primary production at the global scale,” in Phytoplankton Productivity. Carbon Assimilation in Marine and Freshwater Ecosystems. Eds. Williams P. J. Ie B. Thomas D. N. Reynolds C. S. (Oxford, UK: Blackwell Science), 156–186.

[B4] Bethoux J. P. Boukhary E. 1. Ruiz-Pino M. S. Porin D. Copin-Montégut P. (2005). “Nutrient, oxygen and carbon ratios, CO_2_ sequestration and anthropogenic forcing in the Mediterranean Sea,” in The Mediterranean Sea. The Handbook of Environmental Chemistry. Ed. Saliot A. (Berlin/Heidelberg, Germany: Springer), 67–87, ISBN: ISBN-13: 978-3-540-25018-0.

[B5] Bloom A. J. Burger M. Rubio Asensio J. S. Cousins A. B. (2010). Carbon dioxide enrichment inhibits nitrate assimilation in wheat and Arabidopsis. Science 328 (5980), 899–903. 10.1126/science.1186440 20466933

[B6] Borum J. Pedersen O. Kotula L. Fraser M. W. Statton J. Colmer T. D. (2016). Photosynthetic response to globally increasing CO_2_ of co-occurring temperate seagrass species. Plant Cell Environ. 39 (6), 1240–1250. 10.1111/pce.12658 26476101

[B7] Britto D. T. Ruth T. J. Lapi S. Kronzucker H. J. (2004). Cellular and whole-plant chloride dynamics in barley: Insights into chloride-nitrogen interactions and salinity responses. Planta 218 (4), 615–622. 10.1007/s00425-003-1137-x 14663584

[B8] Chen Y. H. Hu L. Punta M. Bruni R. Hillerich B. Kloss B. (2010). Homologue structure of the SLAC1 anion channel for closing stomata in leaves. Nature 467, 1074–1080. 10.1038/nature09487 20981093 PMC3548404

[B9] Cookson S. J. Williams L. E. Miller A. J. (2005). Light-dark changes in cytosolic nitrate pools depend on nitrate reductase activity in Arabidopsis leaf cells. Plant Phys. 138 (2), 1097–1105. 10.1104/pp.105.062349 PMC1150423 15908593

[B10] Cotrufo M. F. Ineson P. Scott A. (1998). Elevated CO_2_ reduces the nitrogen concentration of plant tissues. Glob. Change Biol. 4, 43–54. 10.1046/j.1365-2486.1998.00101.x

[B11] Curtis P. S. Wang X. (1998). A meta-analysis of elevated CO_2_ effects on woody plant mass, form and physiology. Oecologia 113, 299–313. 10.1007/s004420050381 28307814

[B12] Demir F. Horntrich C. Blachutzik J. O. Scherzer S. Reinders Y. Kierszniowska S. (2013). Arabidopsis nanodomain-delimited ABA signaling pathway regulates the anion channel SLAH3. Proc. Natl. Acad. Sci. U.S.A. 110, 8296–8301. 10.1073/pnas.1211667110 23630285 PMC3657796

[B13] Dreyer I. Gomez-Porras J. L. Riaño-Pachón D. M. Hedrich R. Geiger D. (2012). Molecular evolution of slow and quick anion channels (SLACs and QUACs/ ALMTs). Front. Plant Sci. 3, 263. 10.3389/fpls.2012.00263 23226151 PMC3509319

[B14] Duarte C. M. Chiscano C. L. (1999). Seagrass biomass and production: A reassessment. Aquat. Bot. 65, 159–174. 10.1016/S0304-3770(99)00038-8

[B15] Fan X. Jia L. Li Y. Smith S. J. Miller A. J. Shen Q. (2007). Comparing nitrate storage and remobilization in two rice cultivars that differ in their nitrogen use efficiency. J. Exp. Bot. 58 (7), 1729–1740. 10.1093/jxb/erm033 17351248

[B16] Felle H. (1994). The H^+^/Cl^–^ symporter in root-hair cells of *Sinapis alba* . Plant Physiol. 106, 1131–1136. 10.1104/pp.106.3.1131 12232395 PMC159640

[B17] Fernández J. A. García-Sánchez M. J. Felle H. (1999). Physiological evidence from a proton pump at the plasma membrane of the marine angiosperm *Zostera marina* L. J. Exp. Bot. 50, 1763–1768. 10.1093/jxb/50.341.1763

[B18] García-Robledo E. Corzo E. Papaspyrou S. (2014). A fast and direct spectrophotometric method for the sequential determination of nitrate and nitrite at low concentrations in small volumes. Mar. Chem. 162, 30–36. 10.1016/j.marchem.2014.03.002

[B19] García-Sánchez M. J. Jaime M. P. Ramos A. Sanders D. Fernández J. A. (2000). Sodium-dependent nitrate transport at the plasma membrane of leaf cells of the marine higher plant *Zostera marina* L. Plant Phys. 122 (3), 879–885. 10.1104/pp.122.3.879 PMC58924 10712552

[B20] Geiger D. Scherzer S. Mumm P. Stange A. Marten I. Bauer H. (2009). Activity of guard cell anion channel SLAC1 is controlled by drought-stress signaling kinase-phosphatase pair. Proc. Natl. Acad. Sci. U.S.A. 106, 21425–21430. 10.1073/pnas.0912021106 19955405 PMC2795561

[B21] Geiger D. Maierhofer T. Al-Rasheid K. A. Scherzer S. Mumm P. Liese A. (2011). Stomatal closure by fast abscisic acid signaling is mediated by the guard cell anion channel SLAH3 and the receptor RCAR1. Sci. Signal. 4, ra32. 10.1126/scisignal.2001346 21586729

[B22] Hedrich R. Geiger D. (2017). Biology of SLAC1-type anion channels - from nutrient uptake to stomatal closure. New Phytol. 216, 46–61. 10.1111/nph.14685 28722226

[B23] Huber J. L. Huber S. C. Campbell W. H. Redinbaugh M. G. (1992). Reversible light/dark modulation of spinach leaf nitrate reductase activity involves protein phosphorylation. Arch. Biochem. Biophys. 296, 58–65. 10.1016/0003-9861(92)90544-7 1605645

[B24] Jablonski L. M. Wang X. Curtis P. S. (2002). Plant reproduction under elevated CO_2_ conditions: a meta-analysis of reports on 79 crop and wild species. New Phytol. 156, 9–26. 10.1046/j.1469-8137.2002.00494.x

[B25] Kaiser W. M. Spill D. Brendle-Behnisch E. (1992). Adenine nucleotides are apparently involved in the light-dark modulation of spinach-leaf nitrate reductase. Planta 186, 236–240. 10.1007/BF00196253 24186663

[B26] Koch M. Bowes G. Ross C. Zhang X. H. (2013). Climate change and ocean acidification effects on seagrasses and marine macroalgae. Glob. Change Biol. 19 (1), 103–132. 10.1111/j.1365-2486.2012.02791.x 23504724

[B27] Larkum W. D. Orth R. J. Duarte C. M. (2006). Seagrasses: Biology, Ecology and Conservation (Netherlands: Springer).

[B28] Lepoint G. Millet S. Dauby P. Gobert S. Bouquegneau J. M. (2002). Annual nitrogen budget of the seagrass *Posidonia oceanic*a as determined by in situ uptake experiments. Mar. Ecol. Prog. Ser. 237, 87–96. 10.3354/meps237087

[B29] Loladze I. (2014). Hidden shift of the ionome of plants exposed to elevated CO_2_ depletes minerals at the base of human nutrition. ELife 2014 (3), 1–29. 10.7554/eLife.02245 PMC4034684 24867639

[B30] Maierhofer T. Lind C. Hüttl S. Scherzer S. Papenfuß M. Simon J. (2014). A single-pore residue renders the Arabidopsis root anion channel SLAH2 highly nitrate selective. Plant Cell. 26 (6), 2554–2567. 10.1105/tpc.114.125849 24938289 PMC4114951

[B31] Miller A. J. Smith S. J. (1996). Nitrate transport and compartmentation in cereal root cells. J. Exp. Bot. 47 (7), 843–854. 10.1093/jxb/47.7.843

[B32] Miller A. J. Smith S. J. (2008). Cytosolic nitrate ion homeostasis: Could it have a role in sensing nitrogen status? Ann. Bot. 101, 485–489. 10.1093/aob/mcm313 18089584 PMC2710192

[B33] Miller A. J. Zhen R. G. (1991). Measurement of intracellular nitrate concentrations in Chara using nitrate-selective microelectrodes. Planta 184 (1), 47–52. 10.1007/BF00208235 24193928

[B34] Negi J. Matsuda O. Nagasawa T. Oba Y. Takahashi H. Kawai-Yamada M. (2008). CO_2_ regulator SLAC1 and its homologues are essential for anion homeostasis in plant cells. Nature 452, 483–486. 10.1038/nature06720 18305482

[B35] Norby R. J. Wullschleger S. D. Gunderson C. A. Johnson D. W. Ceulemans R. (1999). Tree responses to rising CO_2_ in field experiments: implications for the future forest. Plant Cell Environ. 22, 683–714. 10.1046/j.1365-3040.1999.00391.x

[B36] Olsen J. L. Rouzé P. Verhelst B. Lin Y. C. Bayer T. Collen J. (2016). The genome of the seagrass Zostera marina reveals angiosperm adaptation to the sea. Nature 530 (7590), 331–335. 10.1038/nature16548 26814964

[B37] Planes M. D. Niñoles R. Rubio L. Bissoli G. Bueso E. García-Sánchez M. J. (2015). A mechanism of growth inhibition by abscisic acid in germinating seeds of Arabidopsis thaliana based on inhibition of plasma membrane H^+^-ATPase and decreased cytosolic pH, K^+^, and anions. J. Exp. Bot. 66 (3), 813–825. 10.1093/jxb/eru442 25371509 PMC4321545

[B38] Ravaglioli C. Lauritano C. Buia M. Balestri E. Capocchi A. Fontanini D. (2017). Nutrient Loading Fosters Seagrass Productivity Under Ocean Acidification. Sci. Rep. 7, 13732. 10.1038/s41598-017-14075-8 29062025 PMC5653774

[B39] Raven J. A. Caldeira K. Elderfield H. Hoegh-Guldberg O. Liss P. Riebesell U. (2005). Ocean Acidification due to Increasing Atmospheric Carbon Dioxide (London: The Royal Society). Policy Document 12/05.

[B40] Raven J. A. Beardall J. Giordano M. (2014). Energy costs of carbon dioxide concentrating mechanisms in aquatic organisms. Photosynth. Res. 121, 111–124. 1. 10.1007/s11120-013-9962-7 24390639

[B41] Raven J. A. (1986). “Physiological consequences of extremely small size for autotrophic organisms in the sea,” in Photosynthetic Picoplankton. Eds. Platt T. Li W. K. W. (Ottawa, Canada: Canadian Bulletin of Fisheries and Aquatic Sciences), 1–70.

[B42] Riens B. Heldt H. W. (1992). Decrease of nitrate reductase activity in spinach leaves during a light-dark transition. Plant Physiol. 98, 573–577. 10.1104/pp.98.2.573 16668679 PMC1080228

[B43] Roberts S. K. (2006). Plasma membrane anion channels in higher plants and their putative functions in roots. New Phytol. 169, 647–666. 10.1111/j.1469-8137.2006.01639.x 16441747

[B44] Roelfsema M. R. G. Levchenko V. Hedrich R. (2004). ABA depolarizes guard cells in intact plants, through a transient activation of R- and S-type anion channels. Plant J. 37, 578–588. 10.1111/j.1365-313x.2003.01985.x 14756768

[B45] Roelfsema M. R. G. Hedrich R. Geiger D. (2012). Anion channels: Master switches of stress responses. Trends Plant Sci. 17 (4), 221–229. 10.1016/j.tplants.2012.01.009 22381565

[B46] Romero J. Lee K. S. Pérez M. A. Alcoverro T. (2006). “Nutrients dynamics,” in Seagrasses: Biology, Ecology and Conservation. Eds. Larkum A. W. D. Orth R. J. Duarte C. M. (Netherlands: Springer), 227–254.

[B47] Rubio L. Fernández J. A. (2019). “Seagrasses, the unique adaptation of angiosperms to the marine environment: effect of high carbon and ocean acidification on energetics and ion homeostasis,” in Halophytes and Climate Change: Adaptive Mechanisms and Potential Uses. Eds. Hasanuzzaman M. Shabala S. Fujita M. (Boston M.A.: CAB International), 89–103.

[B48] Rubio L. Linares-Rueda A. García-Sánchez M. J. Fernández J. A. (2005). Physiological evidence for a sodium-dependent high-affinity phosphate and nitrate transport at the plasma membrane of leaf and root cells of *Zostera marina* L. J. Exp. Bot. 56 (412), 613–622. 10.1093/jxb/eri053 15611145

[B49] Rubio L. Belver A. Venema K. García-Sánchez M. J. Fernández J. A. (2011). Evidence for a sodium efflux mechanism in the leaf cells of the seagrass *Zostera marina* L. J. Exp. Mar. Biol. Ecol. 402, 56–64. 10.1016/j.jembe.2011.03.016

[B50] Rubio L. García D. García-Sánchez M. J. Niell F. X. Felle H. H. Fernández J. A. (2017). Direct uptake of HCO_3_ ^–^ in the marine angiosperm *Posidonia oceanica* (L.) Delile driven by a plasma membrane H^+^ economy. Plant Cell Environ. 40, 2820–2830. 10.1111/pce.13057 28815648

[B51] Rubio L. García-Pérez D. García-Sánchez M. J. Fernández J. A. (2018). Na^+^-dependent high-affinity nitrate, phosphate and amino acids transport in leaf cells of the seagrass P*osidonia oceanica* (L.) Delile. Int. J. Mol. Sci. 19 (6), 1570. 10.3390/ijms19061570 PMC6032226 29795043

[B52] Sültemeyer D. Schmidt R. Heinrich P. F. (1993). Carbonic anhydrase in higher plants and aquatic microorganisms. Physiol. Plant 88, 179–190. 10.1111/j.1399-3054.1993.tb01776.x

[B53] Scartazza A. Moscatello S. Gavrichkova O. Buia M. C. Lauteri M. Battistelli A. (2017). Carbon and nitrogen allocation strategy in Posidonia oceanica is altered by seawater acidification. Sci. Total Environ. 607–608, 954–964. 10.1016/j.scitotenv.2017.06.084 28724227

[B54] Schmidt C. Schroeder J. I. (1994). Anion selectivity of slow anion channels in the plasma membrane of guard cells (large nitrate permeability). Plant Physiol. 106, 383–391. 10.1104/pp.106.1.383 12232336 PMC159537

[B55] Schmidt C. Schelle I. Liao Y. J. Schroeder J. I. (1995). Strong regulation of slow anion channels and abscisic-acid signaling in guard-cells by phosphorylation and dephosphorylation events. Proc. Natl. Acad. Sci. U.S.A. 92, 9535–9539. 10.1073/pnas.92.21.9535 11607582 PMC40836

[B56] Siddiqi M. Y. Glass A. D. M. (2002). An evaluation of the evidence for, and implications of, cytoplasmic nitrate homeostasis. Plant Cell Environ. 25, 1211–1217. 10.1046/j.1365-3040.2002.00927.x

[B57] Taub D. R. Wang X. (2008). Why are nitrogen concentrations in plant tissues lower under elevated CO_2_? A critical examination of the hypotheses. J. Integr. Plant Biol. 50 (11), 1365–1374. 10.1111/j.1744-7909.2008.00754.x 19017124

[B58] Taub D. R. Miller B. Allen H. (2008). Effects of elevated CO_2_ on the protein concentration of food crops: A meta-analysis. Glob. Change Biol. 14 (3), 565–575. 10.1111/j.1365-2486.2007.01511.x

[B59] Touchette B. W. Burkholder J. A. M. (2000). Overview of the physiological ecology of carbon metabolism in seagrasses. J. Exp. Mar. Biol. Ecol. 250 (1–2), 169–205. 10.1016/S0022-0981(00)00196-9 10969168

[B60] Trębacz K. Simonis W. Schönknecht G. (1994). Cytoplasmic Ca^2+^, K^+^, Cl^–^, and NO_3_ ^–^ activities in the liverwort *Conocephalum conicum* L. at rest and during action potentials. Plant Phys. 106 (3), 1073–1084. 10.1104/pp.106.3.1073 PMC159633 12232388

[B61] Van Der Leij M. Smith S. J. Miller A. J. (1998). Remobilisation of vacuolar stored nitrate in barley root cells. Planta 205 (1), 64–72. 10.1007/s004250050297

[B62] Williams S. L. (2016). Genomics: From sea to sea. Nature 530, 290–291. 10.1038/nature16869 26814973

[B63] Xue S. Hu H. Ries A. Merilo E. Kollist H. Schroeder J. I. (2011). Central functions of bicarbonate in S-type anion channel activation and OST1 protein kinase in CO_2_ signal transduction in guard cell. EMBO J. 30 (8), 1645–1658. 10.1038/emboj.2011.68 21423149 PMC3102275

[B64] Zeebe R. E. Wolf-Gladrow D. (2001). CO2vin Seawater: Equilibrium, Kinetics, Isotopes. 1st edn (Amsterdam: Elsevier).

[B65] Zhen R. G. Koyro H. W. Leigh R. A. Tomos A. D. Miller A. J. (1991). Compartmental nitrate concentrations in barley root cells measured with nitrate-selective microelectrodes and by single-cell sap sampling. Planta 185 (3), 356–361. 10.1007/BF00201056 24186418

[B66] Zheng X. He K. Kleist T. Chen F. Luan S. (2015). Anion channel SLAH3 functions in nitrate dependent alleviation of ammonium toxicity in Arabidopsis. Plant Cell Environ. 38, 474–486. 10.1111/pce.12389 24944085

